# Intrathecal rituximab for the treatment of Epstein–Barr virus-associated encephalitis

**DOI:** 10.3389/fimmu.2025.1736017

**Published:** 2025-12-11

**Authors:** Aude G. Chapuis, Johnnie J. Orozco, Filippo Milano

**Affiliations:** 1 Translational Science and Therapeutics, Fred Hutchinson Cancer Center, Seattle, WA, United States; 2 Department of Medicine, University of Washington School of Medicine, Seattle, WA, United States

**Keywords:** EBV encephalitis, allogeneic transplant, rituximab, immunosuppression, intrathecal

## Abstract

Epstein–Barr virus (EBV)-associated encephalitis is seen in patients who have undergone allogeneic hematopoietic stem cell transplant and can be associated with significant morbidity and mortality. The mainstay of treatment has been antiviral therapy with nucleoside analogues and reduction of immunosuppression. Here, we describe an adult patient diagnosed with refractory EBV-associated encephalitis within 30 days post-allogeneic transplant successfully treated with intrathecal rituximab, which, to our knowledge, is the first case treated in this manner.

## Introduction

Viral infections of the central nervous system (CNS) can occur in a significant proportion of patients undergoing allogeneic hematopoietic stem cell transplant (HSCT) and contribute significantly to morbidity and mortality (1, 2). The most common pathogens involved include human herpes virus 6 (HHV6), herpes simplex virus (HSV), Epstein–Barr virus (EBV), cytomegalovirus (CMV), varicella zoster virus (VZV), and JC virus (3, 4).

EBV is a double-stranded DNA virus in the gamma herpesvirus family, infecting up to 90% of the worldwide adult population (5). EBV infection typically results in mild and self-limiting infectious mononucleosis syndromes usually in children, adolescents, and young adults (6). However, EBV can be associated with malignant transformation, including B- and T-cell lymphomas and nasopharyngeal carcinoma, as well as post-transplant lymphoproliferative disorder (PTLD) in recipients of HSCT or solid organ transplant (7–10). EBV-associated CNS infection (EBV-CNS) can occur as primary infection or due to reactivation of latent EBV in both HSCT (11, 12) and solid organ transplant patients (13, 14), and can be driven by PTLD. Neurologic sequelae of EBV infection are varied and include meningitis, encephalitis, myelitis, ataxia, cranial or peripheral nerve deficits, seizures, and psychiatric abnormalities including altered mental status and hallucinations (11, 15).

There are no clear guidelines for the treatment of EBV meningitis or encephalitis with paradigms including supportive care, corticosteroids, reduction of immunosuppression, and antivirals including acyclovir and ganciclovir (16). Rituximab, a monoclonal antibody against CD20, is used in the management of PTLD but does not cross the blood–brain barrier. Intrathecal (IT) rituximab has been used in case reports to treat PTLD not responsive to reduction of immunosuppression or systemic rituximab (17, 18) but not encephalitis. We report here, to our knowledge, the first use of IT rituximab for the treatment of EBV-associated encephalitis in an HSCT recipient.

## Case report

A 23-year-old male patient with a history of Philadelphia chromosome-positive B-cell acute lymphoblastic leukemia (Ph+ B-ALL) was referred to our service for HSCT. He had no prior leukemic CNS involvement and had achieved complete remission following induction with hyperCVAD and dasatinib.

Transplant conditioning included myeloablative total body irradiation followed by cyclophosphamide. The patient was CMV, HSV, and *Toxoplasma* seronegative; EBV and VZV seropositive [polymerase chain reaction (PCR) negative]; and ABO type B +. His donor was a human leukocyte antigen (HLA)-matched unrelated female donor, CMV seropositive, and ABO type O +. For graft-versus-host disease (GVHD) prophylaxis, he received tacrolimus and methotrexate. His transplant course was complicated by multiple admissions for neutropenic fever, high-grade mucositis, supraventricular tachycardia, passenger lymphocyte syndrome, and acute skin, gastrointestinal, and liver GVHD requiring steroid treatment at 1 mg/kg on day 9 post-transplant.

**Figure 1:**
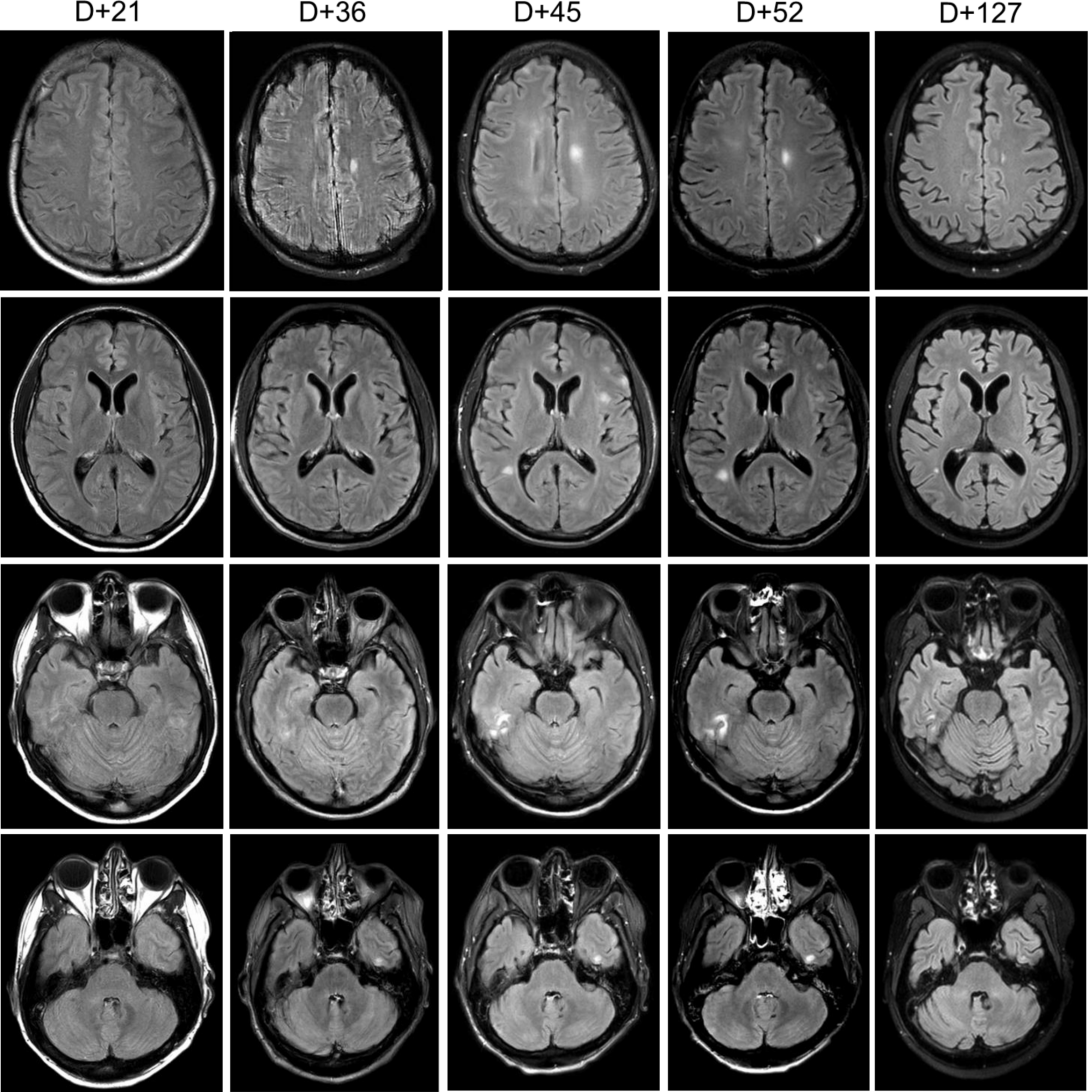
Brain MRI. Serial sections of T2-weighted fluid-attenuated inversion recovery (FLAIR) MRI brain sequences noting evolution areas of cortical enhancement. “D+” denotes day post-HSCT.

On day 20 post-transplant, the patient was admitted with non-neutropenic fever of 39.3°C with associated rigors, headaches, and photophobia. Head computed tomography (CT) (not shown) and brain magnetic resonance imaging (MRI) (**Figure 1**, first column) did not reveal any abnormalities. Cerebrospinal fluid (CSF) studies on day 21 were remarkable for lymphocytic pleocytosis with normal glucose and elevated protein (**Table 1**) with PCR negative for HSV, CMV, EBV, HHV6, VZV, adenovirus, and enterovirus, consistent with aseptic meningitis. CSF flow cytometry was negative for recurrent ALL. While CSF PCR results were pending, he received empiric treatment with treatment-dose acyclovir (10 mg/kg × 3 days) followed by valacyclovir prophylaxis (500 mg BID).

**Table 1:** Selected CSF studies.

Days post-HSCT	Glucose mg/dL (40–80)	Protein mg/dL (15–45)	RBC cells/µL (0–5)	WBC cells/µL (0–5)	Lymphocytes	EBV PCR IU/mL	Intrathecal rituximab
21	63	122	83	14	72%	Undetectable	
34	84	125	123	21	81%	1,400	
38	61	121	362	9	89%	520	+
45	49	95	4	5	72%	70	+
52	48	75	1	5	92%	Undetectable	+
59	59	80	2	6	68%	Undetectable	+

Normal ranges are noted in parentheses.

He remained afebrile for several days though he continued to have headaches, paresthesias, mild expressive aphasia, and hallucinations. On day 31, he redeveloped fever, became hypotensive, and demonstrated witnessed seizure activity, with rigidity and urinary incontinence, followed by decreased responsiveness to stimuli, likely related to a post-ictal state. He was treated with lorazepam, phenytoin, and levetiracetam as well as initiated on broad-spectrum meningitis coverage with ceftriaxone, ampicillin, and acyclovir. A wide differential was considered including meningitis/encephalitis, ALL recurrence, sepsis, tacrolimus toxicity, and posterior reversible encephalopathy syndrome (PRES). Head CT (not shown) did not demonstrate characteristic findings of PRES. Brain MRI (**Figure 1**, second column) noted at least five areas of focal cortical enhancement on T2-weighted FLAIR sequences in the bilateral frontal lobes and left temporal lobe without evidence of leptomeningeal enhancement or hemorrhage. Electroencephalogram was consistent with mild/moderate encephalopathy without epileptic abnormalities. Repeated CSF studies done again on day 34 noted lymphocytic pleocytosis, but this time, CSV EBV PCR was positive (1,400 IU/mL, **Table 1**) while serum EBV levels were undetectable. Additional CSF studies including Bartonella and Toxoplasma PCR and serologies, and bacterial and fungal PCR, were all negative. The bone marrow biopsy was negative for infectious etiology or ALL recurrence. Importantly, throughout this period, immunosuppressive therapy was not tapered given the patient’s ongoing active GVHD.

Given the recurrence and worsening of his neurologic symptoms while already on prophylactic antiviral therapy, we opted to add IT rituximab. He received 15 mg weekly (with dexamethasone premedication) for a total of four doses on days 38, 45, 52, and 59 with CSF EBV undetectable by the administration of the third dose (**Table 1**). Serial MRIs obtained noted an initial increase in lesions (**Figure 1**, third column) with subsequent complete resolution by day 127. Serum EBV levels remained undetectable on all subsequent tests. Of note, IT rituximab was well tolerated, and no adverse effects were noted.

## Discussion

Infectious complications are common in recipients of allogeneic HSCT due to a variety of underlying disease and treatment-related mechanisms, with CNS infections comprising 1%–15% (1, 2). EBV has been shown to be the causative factor in up to nearly 20% of HSCT patients presenting with viral encephalitis with a mortality rate as high as 83% (3). EBV-associated conditions, including febrile illness, encephalitis, and PTLD, were noted to have a 3-year cumulative incidence of nearly 16%, with a median time to onset of 63 days post-transplantation (12). The primary risk factor for EBV-associated CNS disease is immunosuppression, resulting from underlying disease or treatment, and can be seen in recipients of both solid organ and bone marrow transplant and in patients with HIV, lymphoma, and other immunosuppressive disorders. Less commonly, it can occur in the setting of infectious mononucleosis in otherwise immunocompetent patients (6, 11).

Symptoms of EBV-associated CNS disease can range from meningoencephalitis, acute cerebellar ataxia, cranial or peripheral nerve neuropathies, myelitis, seizures, and psychiatric abnormalities (15, 19). Imaging findings can be variable and transient, but often include multifocal intensities in the deep gray nuclei and sub-cortical white matter on T2-weighted and FLAIR MRI (20, 21) as noted in our patient (**Figure 1**). Given the overlapping symptoms and non-specific imaging findings, it is important to distinguish EBV encephalitis from other EBV- or non-EBV-associated etiologies with similar presentation including PTLD, post-infectious myelitis, disease recurrence, and treatment-related toxicities. While EBV encephalitis and CNS-PTLD may present with overlapping neurological symptoms, they differ in biology and management. EBV encephalitis reflects viral inflammation with diffuse MRI changes and inflammatory CSF, whereas CNS-PTLD is an EBV-driven lymphoproliferative process that often produces mass-like lesions and may show atypical lymphoid cells. These distinctions matter clinically, as encephalitis is treated with supportive/antiviral measures, while CNS-PTLD requires PTLD-directed immunotherapy. Our case highlights the importance of this differentiation when EBV DNA is detected in the CNS after transplant. CSF PCR is highly specific and sensitive for detection though false negatives have been reported in HSCT recipients (16).

There are no clear treatment guidelines for EBV encephalitis, though most cases include treatment (often empiric) with antiviral nucleosides, including treatment-level dosing of intravenous acyclovir or ganciclovir, and oral valganciclovir. Reduction of immunosuppression may be efficacious, particularly in PTLD. EBV-associated PTLD is typically treated with the monoclonal CD20 antibody rituximab as monotherapy or as chemoimmunotherapy with or without antiviral agents (10).

Systemic rituximab is noted to have poor CNS penetration with <0.1% of therapeutic serum levels at standard systemic dosing (22). There have been reports of treatment of refractory PTLD with CNS disease and isolated CNS-PTLD with IT rituximab. In one case series (23), two pediatric patients with isolated CNS-PTLD were treated with IT rituximab in combination with either methotrexate or cytarabine, and hydrocortisone. The rituximab/MTX combination was noted to result in CSF clearance and resolution of imaging findings within 4 weeks while the rituximab/cytarabine/hydrocortisone combination resulted in persistent low-level CSF involvement with clinical improvement. These were well-tolerated without significant adverse events. In another study (17), eight pediatric patients diagnosed with CNS-PTLD post-allogeneic HSCT were treated with systemic rituximab in combination with weekly IT rituximab until resolution of clinical disease and serum and CSF EBV clearance. Responses were demonstrated in most patients (five complete and two partial responses), though there was one death due to clinical progression. No serious adverse events were noted, though one subject developed transient seizures with the third dose of IT rituximab requiring benzodiazepine. In a prospective study (18), adult patients with CNS-PTLD with prior failure to systemic therapy received escalating doses of IT rituximab with effective responses observed in eight of nine patients. Two subjects developed transient headaches and cauda equina syndrome following IT rituximab administration, but no other serious adverse events were noted.

In our patient, after ruling out alternative etiologies, we opted to treat with IT rituximab (15 mg weekly) with dexamethasone premedication. Treatment dosing of acyclovir (10 mg/kg IV TID) was also continued and later transitioned to valacyclovir (2 g PO BID). CNS clearance of EBV was achieved following two rituximab administrations (**Table 1**) with a total of four doses administered weekly. Improvement was noted on imaging (**Figure 1**) following third dose administration with complete resolution of imaging findings 4 months following initial onset of symptoms. While CSF EBV levels began to decline prior to IT rituximab administration, likely reflecting some response to systemic antiviral therapy and reduction of immunosuppression, clinical and imaging improvement did not occur until the administration of all four doses. Notably, serum EBV levels remained negative even while CSF EBV was detectable, a discordance previously noted in patients with EBV-associated CNS disease (24). The patient recovered completely without recurrence of neuropsychiatric sequelae or other adverse events.

While our experience was limited to a single patient, when taken in the context of studies of its use in CNS-PTLD, rituximab appears to be a safe, effective, and novel approach for the treatment of antiviral nucleoside-refractory EBV encephalitis.
